# Configurable Modular EEG Classification Framework with Multiscale Features and Ensemble Learning: A Reproducible Evaluation for Schizophrenia Detection

**DOI:** 10.3390/bioengineering13040430

**Published:** 2026-04-07

**Authors:** Xinran Han, Yossef Emara, Alice Zhang, Yi Lin, Yang Zhang

**Affiliations:** 1 Department of Speech-Language-Hearing Sciences, University of Minnesota, Minneapolis, MN 55455, USA; han00610@umn.edu; 2 Department of Computer Science and Engineering, University of Minnesota, Minneapolis, MN 55455, USA; emara004@umn.edu; 3 Wayzata High School, 4955 Peony Ln N, Plymouth, MN 55446, USA; zhangali000@isd284.com; 4 Speech-Language-Hearing Center, School of Foreign Languages, Shanghai Jiao Tong University, Shanghai 200240, China; carol.y.lin@sjtu.edu.cn; 5 National Research Centre for Language and Well-Being, Shanghai 200240, China; 6 Masonic Institute for the Developing Brain, University of Minnesota, Minneapolis, MN 55414, USA

**Keywords:** EEG classification, machine learning, ensemble learning, reproducibility, LOSO validation, data leakage

## Abstract

EEG-based classification of mental disorders has increasingly relied on deep learning models, which are computationally intensive and difficult to interpret, limiting reproducibility and clinical deployment in resource-constrained or cross-site settings. We propose a configurable and modular machine learning framework for EEG-based classification that emphasizes interpretability, flexibility, and rigorous evaluation using schizophrenia detection as a representative use case. Our framework integrates standardized preprocessing, multiscale feature extraction, minimum redundancy–maximum relevance feature selection, and configurable ensemble learning. It also supports multiple validation strategies, including random splits, k-fold cross-validation, and leave-one-subject-out (LOSO), enabling systematic assessment of subject-level generalization. We evaluated the framework on two open EEG datasets: Warsaw IPN (Institute of Psychiatry and Neurology, 19 channels, 250 Hz; 28 subjects) and a Moscow adolescent cohort (16 channels, 128 Hz; 84 subjects). Results show that validation strategy strongly affects model performance. While K-fold validation yielded epoch-level accuracies of 98.06% and 91.47%, LOSO results were much lower: 76.12% (epoch-level) and 82.14% (subject-level) for Dataset 1, and 70.71% (epoch-level) and 77.38% (subject-level) for Dataset 2. These findings demonstrate the risk of overestimated performance due to data leakage and underscore the importance of subject-independent evaluation. Our proposed framework provides a low-complexity, interpretable, and extensible benchmark for reproducible EEG-based machine learning, with interpretable feature representations linked to EEG dynamics and potential applicability to broader neuroengineering and clinical decision-support systems.

## 1. Introduction

Schizophrenia (SZ or SCZ) is a severe and heterogeneous neuropsychiatric disorder affecting approximately 0.3–0.7% of the global population [1]. It is characterized by positive symptoms (e.g., hallucinations and delusions), negative symptoms (e.g., avolition and blunted affect), and persistent cognitive impairments that disrupt daily functioning. Beyond core psychotic symptoms, individuals with SZ exhibit robust deficits in language and social recognition [2], including impaired recognition of facial and vocal emotions [3,4,5,6,7]. Although the behavioral markers are clinically informative, their subjective nature introduces inter-rater variability, limits sensitivity to early and subclinical disease stages, and lacks mechanistic grounding in neural processes. These limitations motivate the need for objective, biologically grounded measures to support scalable diagnosis and monitoring.

Electroencephalography (EEG) provides a promising foundation for such approaches due to its millisecond temporal resolution, non-invasiveness, portability, and sensitivity to neural oscillatory dynamics [8,9]. EEG captures abnormalities in brain rhythms, functional connectivity, and signal complexity that are consistently reported in SZ (e.g., [10,11,12,13]) and reflect dysregulation of large-scale neural networks. However, the high dimensionality, multiscale structure, and noise characteristics of EEG limit the effectiveness of conventional statistical approaches.

Machine learning (ML) has therefore become a key tool for extracting discriminative patterns from EEG data. Recent work has demonstrated strong performance in EEG-based classification for SZ detection, mechanistic interpretation, and clinical outcome prediction (see [14] for a systematic review; see also [15] for a broader review of AI-driven EEG analysis across neurological disorders). More broadly, AI-driven EEG analysis has demonstrated substantial progress across neurological disorders, particularly in clinically established domains such as epilepsy diagnosis and management [16].

### 1.1. Methodological Frameworks in EEG-Based SZ Classification

Most EEG-based SZ classification studies follow a three-stage pipeline: preprocessing, feature extraction, and classification [17,18,19,20]. Preprocessing is essential for reducing contamination from ocular, muscular, and environmental artifacts. Standard procedures include band-pass filtering (commonly 0.5–50 Hz or 1–40 Hz) [19,21,22], re-referencing (e.g., average reference), artifact correction or rejection using Independent Component Analysis (ICA) or related algorithms, and segmentation into epochs for resting-state or event-related analysis [19,20]. Signal normalization (e.g., z-scoring) is often applied to mitigate inter-subject variability (e.g., [23]), although preprocessing choices remain a major source of between-study inconsistency.

EEG abnormalities in SZ manifest across multiple temporal and spatial scales, necessitating complementary feature representations. Feature extraction aims to capture the multiscale neural abnormalities associated with SZ and typically spans several complementary domains: (1) Time-domain features, including statistical descriptors and event-related potentials (ERPs) [24] such as reduced P300 and N100 amplitudes and delayed latencies, reflecting impairments in attention and sensory processing [25,26]. (2) Frequency-domain features, such as power spectral density (PSD) across canonical frequency bands [27], with reduced resting-state alpha power and elevated theta activity, particularly in frontal regions [9,28,29]. (3) Time–frequency features, derived from wavelet or short-time Fourier transforms, capturing transient, non-stationary dynamics that are not visible in static spectral measures [30,31]. (4) Functional connectivity and network features, such as coherence, phase-locking value (PLV), and graph-theoretic metrics, showing widespread dysconnectivity, especially within fronto-temporal and fronto-parietal networks, supporting the dysconnection hypothesis of SZ [32,33,34]. (5) Nonlinear and complexity features, including entropy measures, fractal dimension, and detrended fluctuation analysis (DFA), consistently indicating reduced neural complexity and diminished adaptive flexibility in SZ [35,36]. (6) Emerging representations, such as EEG microstates, model quasi-stable scalp topographies, showing altered temporal dynamics in SZ, particularly in microstates associated with salience and cognitive control networks [37,38,39].

Classification models range from traditional supervised ML to deep learning (DL) and hybrid or ensemble approaches. Traditional classifiers such as Support Vector Machines (SVM) [33,38,40,41,42,43,44], Random Forests (RF) [45,46,47], k-Nearest Neighbors (k-NN or KNN) [48], and Logistic Regression are widely used due to their interpretability, computational efficiency, and suitability for small datasets. These models rely on handcrafted features and often incorporate feature selection methods to reduce dimensionality (e.g., [41,49,50,51]). In contrast, DL architectures, including Convolutional Neural Networks (CNNs) [52,53,54] and Recurrent Neural Networks (RNNs), automatically learn hierarchical spatiotemporal representations directly from raw or minimally processed EEG. For example, hybrid CNN-LSTM (Long Short-Term Memory) architectures have shown state-of-the-art performance in tasks like classification and anomaly detection [55,56,57,58]. While DL models have achieved state-of-the-art performance, they require large labeled datasets, substantial computational resources, and often lack interpretability, posing barriers to clinical adoption [14,59]. Hybrid and ensemble methods attempt to balance these trade-offs by combining complementary feature sets or classifiers and employing techniques such as voting, bagging, or stacking to improve robustness [14,60].

### 1.2. Key Limitations in Existing EEG–ML Studies

A primary source of heterogeneity across EEG-based schizophrenia (SZ) classification studies lies in feature learning strategies (Table 1). Traditional machine learning approaches rely on hand-crafted, domain-informed features, including spectral representations (e.g., FFT-based features), signal decomposition methods (e.g., ICA, RVMD, wavelet transforms), and statistical or texture-based descriptors such as Statistical Local Binary Patterns (SLBP) [19,61,62,63,64,65]. These features are typically classified using conventional algorithms such as SVM, KNN, Linear Discriminant Analysis (LDA), decision trees, and ensemble methods, which remain prevalent across both public (e.g., IPN, Kaggle SCZ) and private datasets [64,66,67]. In contrast, deep learning (DL) models, including Deep ResNets, GoogleNet, and RNN-LSTM architectures, aim to learn hierarchical feature representations directly from minimally processed EEG signals (e.g., [56,68,69,70,71]). Hybrid approaches that combine deep feature extraction with classical classifiers have also been explored to balance representational capacity with limited dataset sizes [68,69]. However, the reported performance gains of DL-based models are often closely tied to data availability and computational resources rather than intrinsic methodological advantages [72].

Substantial variability also arises from differences in EEG preprocessing pipelines. Common practices include bandpass filtering (e.g., Butterworth and FIR filters), artifact removal via ICA, and more advanced decomposition techniques such as RVMD and flexible tunable Q wavelet transform (F-TQWT) [62,63,80,81,82]. These preprocessing choices directly influence feature distributions and learned decision boundaries [83], making reported performance difficult to disentangle from signal conditioning methods and complicating cross-study comparison.

Experimental paradigms further contribute to inconsistency. Task-based EEG paradigms (e.g., auditory oddball or working memory tasks) target specific cognitive processes and event-related potentials such as P300 and N100 [12,84,85,86,87,88], offering mechanistic interpretability but requiring task compliance. Resting-state EEG, which dominates many studies in Table 1, captures intrinsic brain dynamics without task demands [89], improving clinical feasibility but increasing inter-subject variability. These paradigm differences substantially affect both feature representations and neurobiological interpretations.

Validation protocols represent another major limitation. Most studies rely on k-fold cross-validation to estimate internal robustness (e.g., [62,70]), whereas more stringent approaches such as leave-one-subject-out (LOSO) or leave-one-out (LOO) validation are less frequently adopted. All within-dataset validation strategies are susceptible to optimistic bias, particularly in small, high-dimensional EEG datasets. External validation on independent cohorts remains rare [14], limiting confidence in cross-site generalizability and real-world applicability.

Despite these methodological differences, many EEG–ML studies report high classification performance, often exceeding 90% accuracy (Table 1). Rather than indicating a universally superior approach, these results reflect convergent neurophysiological findings in SZ, including reduced alpha-band power, increased theta activity, disrupted fronto-temporal and fronto-parietal connectivity, and decreased signal complexity [8,9,12]. These abnormalities are consistently exploited across diverse modeling frameworks, either explicitly through hand-crafted features or implicitly through learned representations. Emerging work also suggests that EEG-based ML may extend beyond binary classification to cognitive profiling, subtype identification, and symptom prediction [72].

However, significant barriers continue to limit clinical translation. Most studies rely on small, single-site datasets, often private cohorts, reducing robustness and population-level generalizability. Inter-subject variability related to age, medication status, illness duration, and symptom severity further complicates model learning. Combined with high-dimensional feature spaces, limited sample sizes increase the risk of overfitting and inflated performance estimates that fail to replicate across datasets. Furthermore, the lack of standardization in preprocessing, feature extraction, validation protocols, and evaluation metrics undermines reproducibility. Model interpretability remains a critical concern: while DL-based methods capture complex patterns, their black-box nature limits neurobiological insight and clinician trust. Moreover, the predominant focus on binary classification reduces ecological validity, as clinical diagnosis requires discrimination among multiple psychiatric conditions.

A critical and often underrecognized limitation in EEG-based machine learning studies is data leakage arising from improper validation design. Many studies segment EEG recordings into short epochs and randomly split these segments into training and test sets, allowing data from the same subject to appear in both sets. Because epochs from a single subject are highly correlated, models can inadvertently learn subject-specific patterns rather than disease-related features, leading to inflated performance. A recent review by Brookshire et al. (2024) [90] systematically demonstrated this effect, showing that classification accuracy dropped from 99.8% to 53% when switching from segment-based splits to subject-level validation, and reporting that only 27% of reviewed studies adequately avoided subject leakage. This issue is particularly relevant in schizophrenia datasets, where each subject contributes many epochs, further amplifying this bias. Consequently, widely reported accuracies exceeding 95% may substantially overestimate true generalization performance. More reliable evaluation strategies include leave-one-subject-out (LOSO) validation, GroupKFold with subject-level grouping, and external dataset testing, all of which enforce strict separation between training and unseen subjects. But a unifying, standardized framework that systematically integrates these components while enabling reproducible evaluation remains lacking.

Taken together, although EEG-based machine learning approaches show strong potential for SZ classification, their performance remains highly sensitive to methodological choices, dataset characteristics, and evaluation protocols. Ensemble learning offers a principled strategy to mitigate these limitations by integrating complementary classifiers or feature representations, thereby improving robustness in small-to-moderate sample size regimes typical of EEG studies. Despite these advantages, ensemble methods remain underexplored in EEG-based schizophrenia classification (e.g., [61,91]). Many existing approaches rely on computationally intensive or poorly interpretable models, and often lack standardized, reproducible evaluation protocols, particularly with respect to subject-independent validation.

To address these challenges, we propose a configurable and modular EEG classification framework that emphasizes interpretability, flexibility, and reproducible evaluation. The framework integrates standardized preprocessing, multiscale feature extraction, feature selection, and configurable ensemble learning within a unified pipeline, and supports multiple validation strategies for systematic assessment of generalization. Schizophrenia is used in this study as a representative use case to evaluate the framework across datasets and validation settings, rather than as the sole target application.

### 1.3. The Present Study

Building on the framework introduced above, this study implements and evaluates a configurable and interpretable EEG classification pipeline using schizophrenia as a representative use case. The framework performs multiscale feature extraction, including time-domain statistics, spectral band power, wavelet coefficients, and fractal dimension measures per EEG channel, capturing complementary aspects of neural signals across temporal and frequency scales.

To improve robustness while preserving interpretability and computational efficiency, the framework employs an ensemble of traditional classifiers, including Support Vector Machine, K-Nearest Neighbors, Extra Trees, Random Forest, Decision Tree, AdaBoost, and Naive Bayes. Each model is optimized via randomized hyperparameter search and combined through soft voting, averaging class probabilities across models. Feature-level explanations are provided using SHAP (SHapley Additive exPlanations) values to identify the EEG features driving classification.

The framework explicitly supports configurable validation protocols, including conventional cross-validation and leave-one-subject-out (LOSO), as well as stratified cross-dataset testing, ensuring leakage-free and clinically feasible performance estimates. In LOSO, no subject’s data appears in both training and test sets, providing a rigorous estimate of generalization. Cross-dataset evaluation trains on one dataset and tests on an independent dataset collected at a different site with different equipment, offering an out-of-distribution assessment of clinical applicability. By delivering a reproducible, plug-and-play pipeline compatible with standard resting-state EEG, this framework advances EEG-based computational psychiatry from proof-of-concept demonstrations toward scalable and objective tools for schizophrenia detection and neurophysiological analysis.

The remainder of the paper is organized as follows: Section 2 describes the datasets, preprocessing procedures, multidomain feature extraction, nested feature selection via Minimum Redundancy Maximum Relevance (MRMR) and correlation filtering, and the ensemble classification model with LOSO and cross-dataset evaluation protocols. Section 3 presents classification performance on both the primary and independent validation datasets, reporting subject-level and epoch-level accuracy with bootstrap confidence intervals, F1-score, and ROC–AUC metrics. Results are reported for both conventional k-fold cross-validation and leave-one-subject-out (LOSO) validation to enable direct comparison; however, the analysis emphasizes LOSO as a more rigorous and clinically relevant estimate of generalization, while detailed k-fold results are provided in the Supplementary Material. Section 4 discusses the methodological advantages of the framework, including leakage-free cross-validation, subject-level evaluation, and paradigm-agnostic design, as well as clinical implications, limitations, and future directions, including integration with deep learning. Section 5 concludes the paper.

## 2. Materials and Methods

### 2.1. EEG Dataset and Preprocessing

The dataset used in this study was obtained from the public repository of Olejarczyk and Jernajczyk at the Institute of Psychiatry and Neurology in Warsaw [34,89]. This dataset consists of resting-state EEG recordings from 14 healthy controls and 14 patients diagnosed with paranoid schizophrenia (ICD-10: F20.0), with an average recording duration of 1028.7 s per subject. Signals were sampled at 250 Hz from 19 scalp channels: Fp2, F8, T4, T6, O2, Fp1, F7, T3, T5, O1, F4, C4, P4, F3, C3, P3, Fz, Cz, and Pz. The data were stored in EDF format and loaded using the MNE (version 1.11.0) Python library (originally based on the Minimum Norm Estimate method for MEG/EEG analysis) [92]. For preprocessing, the EEG recordings were segmented into 6 s epochs with 2 s overlap, resulting in 7191 epochs, each containing 19 channels with 1500 time points. A bandpass filter (0.5–50 Hz) was applied to remove slow drifts and high-frequency noise, followed by artifact removal using Independent Component Analysis (ICA) via MNE and the Automatic and Tunable Artifact Removal (ATAR) algorithm from the SpKit Python library.

To evaluate the robustness of the proposed framework, a second independent dataset was also used. This dataset was obtained from Moscow University (http://brain.bio.msu.ru/eeg_schizophrenia.htm (accessed on 5 December 2025)) and includes resting-state EEG recordings from adolescents, clinically screened and categorized as healthy controls (*n* = 39) or adolescents exhibiting schizophrenia symptoms (*n* = 45). EEG signals were acquired with 16 scalp electrodes (F7, F3, F4, F8, T3, C3, Cz, C4, T4, T5, P3, Pz, P4, T6, O1, O2) at a sampling rate of 128 Hz, with each recording lasting approximately one minute per subject (7680 samples per channel). The raw EEG data were stored as text files with sequentially concatenated channels. All preprocessing steps, feature extraction procedures, and classification models developed for the primary dataset were directly applied to this independent dataset to assess the robustness and cross-dataset generalization of the proposed approach.

#### ICA and ATAR

Independent Component Analysis ICA is a Blind Source Separation (BSS) method used in signal processing and artifact removal [93] and has been used extensively in the literature surrounding EEG machine learning classification due to its availability in several well established software libraries (e.g., EEGLAB [94], FieldTrip [95], and MNE-Python [92]). This algorithm decomposes EEG signals with multiple channels into independent source signals which can be visually excluded through scalp topography, with newer techniques [96] able to automatically do so when manual selection is unrealistic in large datasets. Despite its popularity, ICA can sometimes be overly aggressive, introducing spurious frequency components or distorting neural signals, which may negatively impact downstream predictive models [97].

**Figure 1:**
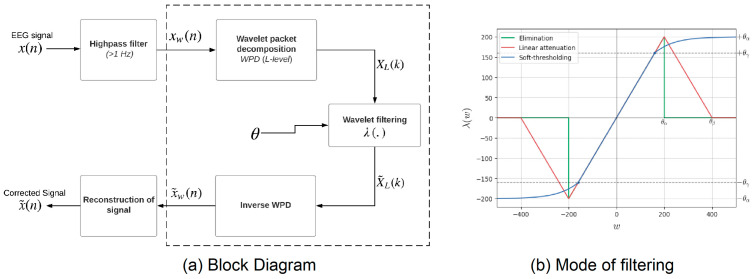
ATAR Algorithm Block Diagram.

A more novel approach that is seldom used is ATAR (Automatic and Tunable Artifact Removal Algorithm) (Figure 1), which uses Wavelet Packet Decomposition to capture temporal patterns in EEG signals [98]. Unlike ICA, ATAR provides a tunable parameter (β) that allows the user to control the aggressiveness of artifact removal, balancing the elimination of ocular and muscle artifacts with the preservation of essential neural signals. In our experiments, ATAR outperformed ICA in improving machine learning model performance, likely due to the controlled artifact removal enabled by setting β = 0.1, which preserved critical EEG features while efficiently mitigating noise.

### 2.2. Feature Extraction

To effectively capture the complex and high-dimensional nature of EEG signals, features were extracted from the time domain and frequency domain, providing a comprehensive representation of neural dynamics [99,100]. Time-domain features included standard statistical metrics such as mean, variance, standard deviation, peak-to-peak amplitude, minimum and maximum signal values, root mean square (RMS), absolute signal difference, skewness, and kurtosis, the latter two capturing asymmetry and peakedness of the signal amplitude distribution. Hjorth parameters—activity (HA), mobility (HM), and complexity (HC)—were also computed, characterizing signal power, the ratio of high-frequency to total signal content, and waveform irregularity, respectively [101]. Frequency-domain features were derived from the power spectral density (PSD) and included band power within the theta (θ, 4–8 Hz), alpha (α, 8–13 Hz), and beta (β, 13–30 Hz) frequency bands, which have been consistently associated with cognitive and pathological states in the EEG literature [102]. Together, these multidomain features provide a rich representation for the ensemble classifier to distinguish between healthy and schizophrenia EEG patterns.

#### 2.2.1. Time Domain Features

Statistical feature extraction from the time domain of the signal has proven to yield promising results when used as features in the task of EEG machine learning classification [103]. The following features were extracted from the signal from every channel:

(a) Sum of Absolute Differences (SAD):

(1) $$SAD=\sum_{i=1}^{N-1}|x_{i+1}-x_{i}|$$

where *n* represents the total number of data points in the time series, $x_{i}$ is the value of the signal at the i-th time point, and $y_{i}$ is the corresponding value of a reference signal or the signal at a previous time point. This feature quantifies the variability and rapid changes in the brain’s electrical activity expressed in time series data.

(b) Root Mean Square (RMS):

RMS captures the overall amplitude and energy contained within a signal, which is correlated with the intensity of neural activity.

(2) $$RMS(x)=\sqrt{\frac{1}{n}\sum_{i=1}^{n}x_{i}^{2}}$$

(c) Hjorth Parameters (Activity, Mobility, and Complexity):

Hjorth Mobility approximates the mean frequency of the signal, defined as the square root of the ratio of the variance of the first derivative to the variance of the original signal.

(3) $$Activity=\sigma^{2}=\frac{1}{N}\sum_{i=1}^{N}(x_{i}-\overset{-}{x}{)}^{2}$$

where $x_{i}$ are the signal values, $\overset{-}{x}$ is the mean of the signal, and *N* is the number of samples.

Hjorth Mobility describes the movement and variation of the spectral components of a signal and describes the mean frequency of the signal as well the proportion of the standard deviation of the power spectrum.

(4) $$Mobility=\sqrt{\frac{\sigma_{\dot{x}}^{2}}{\sigma^{2}}}$$

where $\dot{x}$ is the first derivative of the signal with respect to time, i.e., $\dot{x}(t)=\frac{dx(t)}{dt}$, $\sigma_{x}^{2}$ is the variance of the derivative signal $\dot{x}(t)$, and $\sigma^{2}$ is the variance of the original signal x(t).

Hjorth Complexity computes the similarity between an EEG signal and a pure sine wave which is a measure of complexity and irregularity.

(5) $$Complexity=\frac{Mobility(x^{\prime })}{Mobility(x)}$$

where ${\ddot{x}}_{i}$ are the values of the second derivative of the signal, $\overset{-}{\ddot{x}}$ is the mean of the second derivative, and *N* is the number of samples.

#### 2.2.2. Frequency Domain Features

Frequency-domain feature extraction plays a crucial role in EEG signal analysis, as it provides insight into how signal power is distributed across different frequency bands, which can be associated with specific cognitive states or neurological conditions [98]. To characterize these frequency-domain properties, the power spectral density (PSD) of each EEG channel was computed using Welch’s method. This approach estimates the PSD by dividing the signal into overlapping segments, applying a window function to each segment, performing a Fast Fourier Transform (FFT) on each window, and averaging the resulting spectra across all segments. Welch’s method is particularly well suited for EEG analysis because it reduces variance in the spectral estimate and is effective for handling the non-stationary nature of EEG signals.

Formally, given a signal x[n] of length N, the signal is divided into K overlapping segments, each of length M. For each segment, a periodogram is computed as

(6) $$P_{k}(\omega )=\frac{1}{M}{\left|\sum_{n=(k-1)M+1}^{kM}x[n]e^{-j2\pi nk/N}\right|}^{2}$$

where *k* = 1, 2, …, K, N represents the total length of the signal, K denotes the number of segments, M is the length of each segment, and ω corresponds to the frequency variable.

The final PSD estimate is then obtained by averaging the individual periodograms across all segments:

(7) $$P(\omega )=\frac{1}{K}\sum_{k=1}^{K}P_{k}(\omega )$$

Once the PSD was estimated, band power features were computed by calculating the area under the PSD curve within specific frequency ranges. In particular, power values were extracted for the Delta (δ), Theta (θ), Alpha (α), and Beta (β) frequency bands. In addition to band powers, spectral entropy and other statistical measures were derived from the PSD of each EEG channel to capture the complexity and distribution of spectral content. Finally, additional statistical features listed in Table 1, including the mean, skewness, and kurtosis of the frequency-domain representation, were also extracted to further characterize the spectral properties of the EEG signals.

### 2.3. Feature Selection

To reduce the high dimensionality of the feature space and mitigate overfitting in machine learning models, multiple feature selection strategies were systematically evaluated [104]. Initially, low-variance filtering was applied to remove features with minimal variability across samples, as these features are unlikely to contribute meaningful discriminative information. Next, univariate feature selection using mutual information (MI) was employed, quantifying the dependency between each feature X and the class label Y as:

(8) $$I(X;Y)=\sum_{y\in Y}\sum_{x\in X}p(x,y)\log\left(\frac{p(x,y)}{p(x)p(y)}\right)$$

where *X* denotes the feature and *Y* the class label and x,y represents specific values that the variables *X* and *Y* can take.

Mutual information effectively identifies features that provide the most information about class distinctions, capturing both linear and nonlinear relationships [104].

Following MI-based selection, Recursive Feature Elimination (RFE) was applied using tree-based models to iteratively remove the least informative features, retaining only those that contribute most to model performance [105]. RFE leverages the inherent feature importance scores of tree ensembles, providing a robust and model-aware approach to dimensionality reduction. In parallel, an external tool-based approach using the Featurewiz library was evaluated, implementing a Minimum Redundancy Maximum Relevance (MRMR) criterion [106]. MRMR selects features that are highly relevant to the target variable while minimizing redundancy across the feature set, ensuring a compact yet informative representation. Across multiple experiments, MRMR consistently outperformed alternative strategies and was therefore adopted for all subsequent analyses, reducing the feature set to 50 features per epoch, which balanced model complexity with predictive performance.

### 2.4. Classification Models and Evaluation Protocol

#### 2.4.1. Baseline Models and Conditional Hyperparameter Optimization

To identify optimal features and classifiers, we initially evaluated the following models: Support Vector Machine (SVM), K Nearest Neighbors (KNN), Gradient Boosting, Extra Trees, Random Forest, Decision Trees, Logistic Regression, AdaBoost, Naive Bayes, and a Multilayer Perceptron (MLP). Hyperparameters were not tuned initially, and evaluation was conducted using various sets of feature arrays. A 50–50 random training-testing split was used to evaluate the accuracy of every model on the features array.

#### 2.4.2. Ensemble Learning via Voting and Stacking

Voting and Stacking Ensemble methods have a rich history of usage in machine learning [91], as they enable the aggregation of predictions from multiple models to leverage their individual strengths while mitigating their weaknesses. In this study, Support Vector Machines (SVM), k-Nearest Neighbors (KNN), Gradient Boosting, and Extremely Randomized Trees were employed as base learners within both voting and stacking ensemble frameworks. These ensemble approaches integrate predictions from the individual models using different strategies to improve overall predictive performance. Specifically, the voting classifier combines model outputs either through a hard majority voting scheme or through a soft voting mechanism that averages the predicted class probabilities across all classifiers. In addition, a stacking approach was implemented, in which a logistic regression meta-classifier learns to capture more complex patterns in the data by using the predictions of the base models as input features.

#### 2.4.3. Evaluation Protocol

Classification was performed using multiple machine learning models, including Support Vector Machines (SVM), k-Nearest Neighbors (KNN), Gradient Boosting, Random Forests, and Extremely Randomized Trees. For SVM, a radial basis function (RBF) kernel was used:

(9) $$K(x_{i},x_{j})=\exp\left(-\gamma \|x_{i}-x_{j}{\|}^{2}\right)$$

where $x_{i}$ and $x_{j}$ represent the feature vectors of the i-th and j-th samples, respectively, ∥⋅∥ denotes the Euclidean distance between the two feature vectors, and γ is a kernel parameter that controls the width of the Gaussian function, determining how far the influence of a single training example reaches. The RBF kernel measures the similarity between two samples: the closer $x_{i}$ and $x_{j}$ are in feature space, the higher the kernel value, with a maximum of 1 when they are identical.

To ensure rigorous and leakage-free evaluation, model performance was primarily assessed using leave-one-subject-out (LOSO) cross-validation, in which all data from a single subject are held out for testing while the remaining subjects are used for training. This process is repeated for each subject, ensuring strict separation between training and testing data at the subject level and providing a realistic estimate of subject-independent generalization.

For comparison with conventional practices, k-fold cross-validation was also performed using random partitioning of EEG epochs. Data were split into training and testing sets using a 50/50 split. Within the training set, five-fold cross-validation was used for hyperparameter tuning, and all model performance metrics were evaluated on held-out test data, including accuracy, precision, recall, F1-score, and ROC-AUC for binary classification [107]. For ensemble models, both predictive accuracy and confidence calibration were examined [108]. Final performance metrics were reported on held-out test data only.

However, because the conventional k-fold validation may allow data from the same subject to appear in both training and test sets, potentially leading to optimistic bias, k-fold results are reported primarily for reference, with detailed results provided in the Supplementary Material.

Within each training fold (for both LOSO and k-fold settings), hyperparameter optimization was performed using nested cross-validation to prevent information leakage. Model performance was evaluated using accuracy, precision, recall, F1-score, and receiver operating characteristic area under the curve (ROC–AUC) for binary classification [107]. In addition, both epoch-level and subject-level performance metrics were computed, where subject-level predictions were obtained via aggregation of epoch-level outputs.

These evaluation procedures enable systematic comparison between conventional and subject-independent validation strategies while mitigating overfitting and providing a more reliable estimate of real-world model performance.

## 3. Results

### 3.1. Performance on Dataset 1

The proposed ensemble-based classification framework achieved strong performance on Dataset 1 (see Supplementary Material for more details), with a test accuracy of 98.06%. Both macro-averaged and weighted F1-scores were approximately 0.98, indicating balanced performance across classes. Out of 7206 test epochs, only 140 were misclassified, reflecting robust prediction at the epoch level.

Under LOSO cross validation, which provides a stringent, subject-independent estimate of generalization, the framework achieved an epoch-level accuracy of 76.12% and a subject-level accuracy of 82.14% (23 out of 28 subjects correctly classified). The 95% bootstrap confidence interval for subject-level accuracy was [67.86%, 96.43%] (2000 bootstrap iterations), reflecting meaningful uncertainty due to the modest cohort size. Macro-averaged precision, recall, and F1-score were 0.759, 0.758, and 0.759, respectively, indicating balanced performance across classes. See Table 2 for detailed metrics.

Wilcoxon signed-rank testing with Benjamini–Hochberg correction confirmed that the ensemble classifier significantly outperformed the individual random forest baseline across LOSO folds (mean accuracy: 0.778 ± 0.017 vs. 0.756; W = 0.0, *p* < 0.001, BH-corrected), supporting the benefit of combining complementary base learners even under strict cross-subject evaluation.

**Figure 2:**
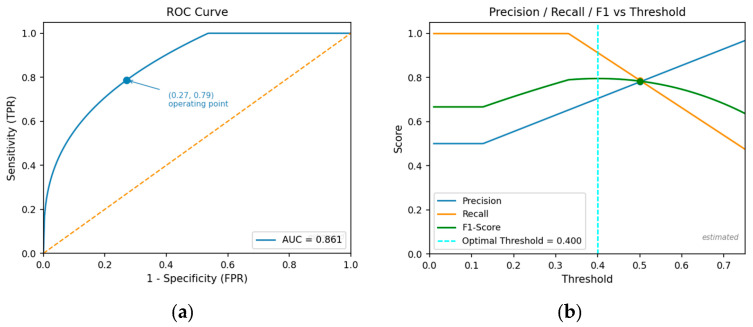
(**a**) ROC curve for Dataset 1 showing the trade-off between sensitivity (True Positive Rate) and 1-specificity (False Positive Rate) for the classifier. The dot indicates the selected operating point (threshold), and the dashed line represents chance-level performance (AUC = 0.5). (**b**) Precision, Recall, and F1-Score plotted against different classification thresholds for Dataset 1. The dashed line indicates the optimal decision threshold (0.400), and the dot marks the corresponding precision, recall, and F1-score values at that threshold.

We used the Receiver Operating Characteristic (ROC) curve, a standard method for evaluating the diagnostic performance of a binary classifier by illustrating the trade-off between the True Positive Rate (sensitivity) and the False Positive Rate (1—specificity) across varying decision thresholds. Figure 2a presents the ROC curve for the proposed model on Dataset 1 under LOSO evaluation. The estimated Area Under the Curve (AUC) of approximately 0.83 reflects solid discriminative ability between schizophrenia and healthy control groups under strict cross-subject conditions. While this represents a substantial reduction relative to the non-LOSO AUC of 0.998 reported previously, it provides a more honest and conservative estimate of the model’s expected performance on unseen subjects. The sharp initial rise of the curve demonstrates that meaningful sensitivity is achievable at relatively low false positive rates. Figure 2b plots precision, recall, and F1-score as functions of the classification threshold, identifying an optimal operating point that maximizes F1-score. These figures collectively characterize both the model’s discriminative capacity and the flexibility to adjust the decision boundary for clinical requirements.

These findings indicate that tree-based ensembles are well-suited to EEG-based SZ classification, providing robust and balanced performance at the epoch level. However, generalization to new subjects and independent datasets remains challenging, highlighting the importance of subject-independent evaluation and further external validation.

### 3.2. Performance on Dataset 2

When evaluated under conventional testing (see Supplementary Material for details), the proposed ensemble framework achieved a test accuracy of 91.47% on Dataset 2. Both macro-averaged and weighted F1-scores were approximately 0.91, indicating balanced classification across classes despite the dataset’s greater heterogeneity. The healthy control group achieved a precision of 92.02% and a recall of 88.29%, while the schizophrenia (SZ) group exhibited a higher recall of 93.97% and a precision of 91.07%. Out of 504 test samples, 43 were misclassified, with errors distributed relatively symmetrically across classes. The modest reduction in conventional performance compared to Dataset 1 reflects the increased variability in Dataset 2 rather than model instability.

Under LOSO cross-validation, the accuracy reduced to 70.71% (epoch level) and 77.38% (subject level) (see Table 3 for performance metrics). The 95% bootstrap confidence interval for subject-level accuracy was [67.86%, 85.71%] (*n* = 84, 2000 bootstrap iterations), reflecting uncertainty inherent in a modestly sized cohort. Macro-averaged precision, recall, and F1-score were 0.706, 0.704, and 0.706, respectively. These LOSO results, while lower than conventional evaluation metrics, demonstrate the model’s robustness across datasets and emphasize the importance of subject-independent validation for realistic performance estimation.

**Figure 3:**
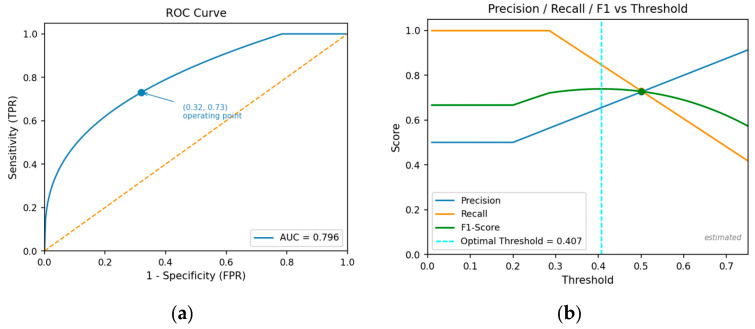
(**a**) Receiver Operating Characteristic (ROC) curve for the proposed classification model evaluated on Dataset 2. The dot indicates the selected operating point (threshold), and the dashed line represents chance-level performance (AUC = 0.5). (**b**) Precision, recall, and F1-score plotted against classification thresholds for the proposed model on Dataset 2. The dashed line indicates the optimal decision threshold (0.407), and the dot marks the corresponding precision, recall, and F1-score values at that threshold.

Figure 3a shows the ROC curve for the LOSO-evaluated model on Dataset 2, illustrating trade-offs between sensitivity and specificity across decision thresholds. The area under the curve (AUC) indicates solid discriminative ability despite cross-subject evaluation. Figure 3b plots precision, recall, and F1-score as functions of the classification threshold, enabling selection of an optimal operating point for potential clinical applications.

These results confirm that ensemble methods maintain relatively stable and balanced performance across heterogeneous EEG datasets, while highlighting the realistic reduction in accuracy under subject-independent evaluation. This underscores the importance of LOSO validation for reliable cross-subject generalization.

### 3.3. Computational Complexity Performance

To assess the practical feasibility of the proposed ensemble framework, we measured both runtime and memory usage during model training and evaluation (Table 4). Across a typical LOSO fold on Dataset 1, the complete pipeline, including preprocessing, multiscale feature extraction, feature selection, and ensemble training, required approximately 10 min per fold. Peak memory usage during training was 17 GB, primarily attributable to storing multiscale feature matrices and model parameters for multiple classifiers in the ensemble.

These results indicate that the proposed framework is computationally efficient relative to deep learning–based EEG approaches, which often require hours of training on GPUs and substantially larger memory footprints. The moderate runtime and memory requirements make the pipeline feasible for deployment in resource-constrained environments, such as clinical or portable EEG setups, and for repeated evaluations across multiple subjects or datasets.

Importantly, the modular design of the framework allows further optimization: individual classifiers can be selectively included or excluded, and feature dimensionality can be adjusted without substantially compromising performance. This flexibility provides a scalable and practical solution for both research and clinical applications of EEG-based schizophrenia classification.

## 4. Discussion

This study presents a modular and interpretable EEG classification framework that integrates tunable artifact suppression, multidomain feature extraction, and ensemble learning. Evaluated under a stringent LOSO protocol, our proposed approach reached a subject-level accuracies of 82.14% and 77.38% on the two schizophrenia datasets, respectively. These results are substantially lower than the very high accuracies using the conventional k-fold validation method. Note that the above 90% (even > 99%) accuracy are frequently reported in prior EEG-based schizophrenia classification studies (Table 1), which shows the critical influence of validation strategy to prevent data leakage and motivates a closer examination of validation methodologies.

### 4.1. Importance of Subject-Level Holdout

A central methodological limitation in EEG-based classification arises from the choice of data partitioning. EEG recordings are segmented into multiple epochs per subject, which are highly correlated. Many prior studies applied conventional k-fold cross-validation or random splitting at the epoch level, inadvertently allowing data from the same subject in both training and test sets. This subject-level data leakage inflates performance estimates by allowing models to learn individual-specific patterns rather than disease-related features [90]. This distinction is particularly important in schizophrenia EEG classification. As shown earlier, a large proportion of prior studies (Table 1) have relied on epoch-level validation schemes, often reporting very high accuracies (>90%, in some studies even >99%), but without enforcing strict subject-level separation (e.g., [61,68,69]). In contrast, only a limited number of studies have explicitly adopted subject-level LOSO evaluation paradigms [77,109]. For example, related study by Wu et al. using a recurrent auto-encoder achieved 81.8% accuracy with LOSO validation [109], which is slightly lower than our 82.14% subject-level accuracy on the same dataset.

These findings clearly demonstrate the impact of validation choice. While k-fold validation yielded very high accuracies, performance decreased substantially under a strict Leave-One-Subject-Out protocol that avoids data leakage. This discrepancy reflects the removal of subject-specific information overlap and provides a more realistic estimate of model performance when applied to unseen individuals.

These findings underscore the critical importance of methodological rigor in EEG-based machine learning, particularly the adoption of leakage-free, subject-level validation protocols. Without such rigor, reported performance may not reflect clinically meaningful generalization, limiting the translational value of these models. By combining a configurable feature-based pipeline with a strictly enforced LOSO evaluation strategy, this study provides a more reliable and transferable benchmark for future EEG-based classification research.

### 4.2. Methodological Contributions: A Configurable and Extensible Pipeline

A central contribution of this work is the design and validation of a modular, end-to-end configurable pipeline that integrates data preprocessing, feature engineering, and ensemble decision-making into a single reproducible framework. Rather than optimizing a fixed architecture for a specific dataset, our pipeline is explicitly designed for adaptability: preprocessing strength, feature composition, and classifier choice can each be independently adjusted without altering the broader experimental logic.

The pipeline’s configurability begins at the preprocessing stage. The framework incorporates Automatic and Tunable Artifact Removal (ATAR), introducing an explicit control parameter (β) that enables adaptive suppression of ocular and muscle artifacts while preserving diagnostically relevant neural signals. This tunable preprocessing mechanism represents a form of computational intelligence at the signal-conditioning level, allowing robustness to be explicitly controlled. Our results indicate that ATAR improves signal quality and downstream classification performance, particularly in datasets with substantial inter-subject variability, distinguishing this approach from standard ICA-based pipelines commonly used in prior studies [61,63,75].

Feature extraction is similarly architected for flexibility. Rather than committing to a narrow domain, our pipeline draws on complementary representations across time and frequency domains, capturing non-Gaussian, nonlinear, and non-stationary properties of neural signals that single-domain approaches systematically miss. Critically, these feature extraction and selection modules are independently substitutable: alternative feature families or selection criteria could be applied without modifying the upstream preprocessing or downstream classification stages. This modularity is what makes the framework genuinely configurable rather than merely parameter-rich.

To manage the resulting high-dimensional feature space, we conducted a systematic evaluation of feature selection strategies, demonstrating that the Minimum Redundancy–Maximum Relevance (MRMR) criterion is particularly effective in retaining discriminative information while minimizing redundancy. This feature-space optimization is critical for stabilizing decision boundaries in small-to-moderate EEG datasets and constitutes an important methodological contribution beyond ad hoc feature pruning.

At the classification stage, the pipeline provides a soft-voting ensemble that aggregates predictions from complementary base learners, consistently outperforming individual classifiers by reducing variance and mitigating overfitting. Importantly, the ensemble architecture is itself a configurable component: base models can be substituted or extended depending on dataset size, computational constraints, or clinical requirements.

Together, our framework’s explicit configurability positions the pipeline not merely as a classification tool for the schizophrenia datasets evaluated here, but as a reproducible and transferable benchmark methodology for EEG-based classification more broadly.

### 4.3. Interpretability, Reliability, and Translational Impact

Interpretability is a critical requirement for clinical decision-support systems and a key limitation of deep learning-based EEG analysis. While CNNs and RNNs can achieve high accuracy [68,69], their latent representations are difficult to map to neurophysiological mechanisms, limiting clinician trust and translational potential. In contrast, our framework maintains transparency across all processing stages, from artifact suppression to feature extraction and ensemble decision-making.

By relying on physiologically meaningful features, our framework enables direct interpretation of classification outcomes in terms of known neural biomarkers. Feature importance analysis revealed consistent contributions from band-power alterations over central regions and elevated entropy measures, aligning with established findings of disrupted oscillatory activity, altered sensorimotor processing, and reduced neural complexity in schizophrenia [61,63,75]. This convergence between computational outcomes and neurophysiological theory enhances both scientific validity and clinical trust.

The confidence-aware ensemble mechanism further improves reliability by mitigating low-certainty predictions, an important consideration for clinical deployment. Unlike opaque end-to-end models, the modular and controllable nature of the proposed framework allows practitioners to adjust preprocessing strength, feature dimensionality, and decision thresholds to accommodate variable EEG quality. Such controllability is essential for handling real-world data variability and supports regulatory and translational requirements for explainable AI systems.

Importantly, interpretable features also support biomarker discovery and clinical translation. By identifying EEG patterns consistently associated with schizophrenia, the framework can inform patient stratification, monitoring of disease progression, and targeted interventions, such as neurofeedback or neuromodulation protocols aimed at normalizing oscillatory activity in affected cortical regions. Unlike purely predictive deep learning models, our approach provides actionable insights that bridge computational performance with clinical relevance.

### 4.4. Limitations and Future Directions

Despite the generalizability demonstrated by the proposed framework, several limitations should be considered. First, dataset 1 consisted of only 14 patients with schizophrenia and 14 healthy controls, which may limit statistical power and the generalizability of the results. In future studies, larger and more diverse cohorts are necessary to fully establish clinical reliability.

Second, the Automatic and Tunable Artifact Removal (ATAR) algorithm, while effective in enhancing signal quality, has not been systematically optimized across different EEG populations or recording protocols. Systematic sensitivity analyses and standardized parameter selection strategies will be important to improve reproducibility and facilitate broader adoption. Nonetheless, the tunable nature of ATAR provides a clear advantage over fixed preprocessing pipelines, enabling adaptive signal conditioning tailored to varying EEG quality.

Third, ensemble classifiers improved accuracy over single classifiers but introduce additional computational overhead, which may limit real-time or resource-constrained clinical applications. While the overall framework remains substantially more efficient than deep learning models, future work should investigate lightweight ensemble designs, model pruning, or dynamic ensemble selection to further reduce inference time and support near-real-time deployment in constrained clinical environments.

Beyond these framework-specific considerations, EEG-based schizophrenia classification continues to face systemic challenges. Public datasets remain limited in size and heterogeneous in electrode layouts, recording protocols, and participant characteristics, complicating cross-study comparison and algorithm benchmarking [61,63,68,75]. Additionally, symptom heterogeneity and class imbalance remain major obstacles to developing universally applicable classifiers.

Future research should explore multimodal bioengineering frameworks that integrate EEG with behavioral and cognitive performance measures (e.g., [4,5]). Hybrid learning strategies, including graph-based models, attention mechanisms, or transformer-based architectures, could exploit complementary information across modalities while preserving interpretability through structured feature representations [110,111]. Such approaches could improve diagnostic precision, enable symptom profiling, and support individualized monitoring and intervention, particularly in disorders where neural dysfunction and observable behavior are tightly coupled. In this context, emerging frameworks such as AI-driven digital twin models have been proposed to enable personalized and dynamically optimized EEG analysis, representing a promising direction for precision neuropsychiatric assessment [112]. Key challenges include temporal alignment, heterogeneous feature fusion, and maintaining explainability, though advances in sensor fusion and interpretable AI provide encouraging directions [113].

Furthermore, progress toward clinical translation will require coordinated efforts in open data sharing, standardized preprocessing, rigorous external validation, and systematic evaluation of ensemble and hybrid models. By prioritizing controllability, interpretability, and data efficiency, future systems can move beyond proof-of-concept classification toward reliable, clinically actionable decision-support tools that complement traditional behavioral assessment in schizophrenia and other disorders.

## 5. Conclusions

This study provides a configurable and methodologically rigorous EEG classification framework for schizophrenia using ensemble machine learning and subject-level validation. The proposed approach achieved an overall accuracy of 82.14% (subject-level) and F1-score of 0.759 on the tested schizophrenia dataset 1, and 77.38% accuracy on dataset 2, demonstrating relatively stable performance across independent cohorts. Consistent with other ML studies on the data leakage problem, our findings demonstrate the importance of a strict Leave-One-Subject-Out (LOSO) protocol, ensuring that all epochs from a given individual are strictly separated between training and testing, which provides a more realistic benchmark of model performance in real-world clinical scenarios. In addition, we introduce a modular, configurable, and interpretable EEG classification pipeline, integrating multidomain feature extraction, tunable artifact suppression, and ensemble learning within a transparent and computationally efficient framework. This design enables adaptability to varying datasets, supports reproducibility, and enhances interpretability, to bridge the gap between predictive performance and neurophysiological understanding. Overall, our work contributes to ongoing efforts to improve the rigor, transparency, and comparability of EEG-based psychiatric classification studies, providing a reproducible benchmark and a practical framework that can inform future research and support the development of clinically actionable EEG-based decision-support tools.

## Figures and Tables

**Table 1:** Summary of schizophrenia classification studies with EEG signals.

Study	Accuracy (%)	Preprocessing Method	Model Used	Database
[61]	99.25	Fast Fourier transform (FFT) and statistical feature	SVM, KNN, Boosted Tree (BT), and Decision Tree (DT)	IPN and Kaggle SCZ dataset
[68]	99.23	Average filtering	Deep ResNets, softmax layer and deep features with SVM	Kaggle SCZ dataset
[69]	98.84	Average filtering	GoogleNet and deep features, SVM	Kaggle SCZ dataset
[63]	89.21	EEGLAB and ICA	KNN, LDA, and SVM	Private
[62]	92.93	Robust variational mode decomposition (RVMD)	Optimized extreme machine classifier	Kaggle SCZ dataset
[37]	90.93	Bandpass filter	SVM and Bayesian optimization	IPN
[70]	98	Dimensionality reduction algorithm	RNN-LSTM	LNNCI [73]
[50]	92.17	ICA	Black Hole (BH) optimization and SVM	IPN
[74]	91.66	Symmetrically Weighted local binary patterns (SLBP) and correlation	Logit Boost classifier	LNNCI, MHRC
[75]	93.9	Finite impulse response (FIR) filter	KNN, Artificial Neural Network (ANN), and SVM	Kaggle SCZ dataset
[67]	99.91	CGP17Pat and Iterative neighborhood component analysis (INCA)	KNN	IPN
[41]	92.91	Butterworth filter and Segmentation	DT, Linear-Discriminant Analysis (LDA), KNN, Probabilistic-Neural-Network (PNN), and SVM	IPN
[76]	71	Butterworth filter and Independent component analysis (ICA)	LDA, and Rule-based classifier	Private
[77]	72.4	Digital filters and ICA	KNN, LDA, SVM	Private
[78]	93.4	Spatial filters and Bandpass filter	SVM, Bayesian LDA, Gaussian NB, KNN, Adaboost, and Radial basis function (RBF)	Private
[79]	91.39	Flexible tunable Q wavelet transform (F-TQWT)	Flexible least square support vector machine (F-LSSVM) classifier and grey wolf optimization (GWO) algorithm	Kaggle SCZ dataset

**Table 2:** Performance Metrics of Dataset 1.

Class	Precision	Recall	F1-Score	Support
Control	0.738	0.729	0.734	6505
SZ	0.780	0.787	0.783	7906
Macro Avg	0.759	0.758	0.759	14,411
Weighted Avg	0.761	0.761	0.761	14,411

Note: Recall is class-specific: the SZ recall measures how well the model detects schizophrenia (sensitivity), and the Control recall measures how well healthy subjects are correctly identified (specificity).

**Table 3:** Performance Metrics of Dataset 2.

Class	Precision	Recall	F1-Score	Support
Control	0.686	0.680	0.683	1170
SZ	0.725	0.730	0.728	1350
Macro Avg	0.706	0.705	0.706	2520
Weighted Avg	0.707	0.707	0.707	2520

**Table 4:** Computational Complexity Metrics.

Metric	Value
Training time (per fold)	10 min
Memory usage	17 GB

## Data Availability

The code used for EEG preprocessing, feature extraction, and machine learning analyses in this study is publicly available on GitHub at https://github.com/zhang470 (accessed on 2 April 2026). The two EEG datasets analyzed in this study are publicly available from their original sources and are cited in the manuscript.

## Supplementary Materials

The following supporting information can be downloaded at: https://www.mdpi.com/article/10.3390/bioengineering13040430/s1, Table S1. Performance Metrics of Dataset 1. Table S2. Performance of Different Classification Methods on Dataset 1. Table S3. Performance Metrics of Dataset 2. Table S4. Performance of Different Classification Methods on Dataset 2. Figure S1. (a) ROC curve for Dataset 1 showing the trade-off between sensitivity (True Positive Rate) and 1-specificity (False Positive Rate) for the classifier. The curve demonstrates excellent performance with an Area Under the Curve (AUC) of 0.998, indicating high discriminative ability. (b) Precision, Recall, and F1-Score plotted against different classification thresholds for Dataset 1. The plot identifies the optimal threshold at 0.434, where the F1-Score is maximized, balancing precision and recall effectively. Figure S2. (a) Receiver Operating Characteristic (ROC) curve for the proposed classification model evaluated on Dataset 2. (b) Precision, recall, and F1-score plotted against classification thresholds for the proposed model on Dataset 2.

## Abbreviations

The following abbreviations are used in this manuscript:

ADHD	Attention-Deficit/Hyperactivity Disorder
AdaBoost	Adaptive Boosting
ANN	Artificial Neural Network
ATAR	Automatic and Tunable Artifact Removal
AUC	Area Under the Curve
BH	Black Hole (optimization algorithm)
BSS	Blind Source Separation
BT	Boosted Tree
CGP17Pat	Cartesian Genetic Programming 17 Pattern
CNN	Convolutional Neural Network
CV	Cross-Validation
DFA	Detrended Fluctuation Analysis
DL	Deep Learning
DT	Decision Tree
EDF	European Data Format
EEG	Electroencephalography
ERP	Event-Related Potential
FFT	Fast Fourier Transform
FIR	Finite Impulse Response
F-LSSVM	Flexible Least Squares Support Vector Machine
F-TQWT	Flexible Tunable Q Wavelet Transform
GWO	Grey Wolf Optimization
HA	Hjorth Activity
HC	Hjorth Complexity
HM	Hjorth Mobility
ICA	Independent Component Analysis
ICD-10	International Classification of Diseases, 10th Revision
INCA	Iterative Neighborhood Component Analysis
IPN	Institute of Psychiatry and Neurology
KNN	k-Nearest Neighbors
LDA	Linear Discriminant Analysis
LNNCI	Laboratory for Neurophysiology and Neuro-Computer Interfaces
LOO	Leave-One-Out
LOSO	Leave-One-Subject-Out
LSTM	Long Short-Term Memory
MI	Mutual Information
ML	Machine Learning
MLP	Multilayer Perceptron
MHRC	Mental Health Research Center
MNE	Minimum Norm Estimation (Python library)
MRMR	Minimum Redundancy Maximum Relevance
NB	Naive Bayes
PLV	Phase-Locking Value
PNN	Probabilistic Neural Network
PSD	Power Spectral Density
RBF	Radial Basis Function
RF	Random Forest
RFE	Recursive Feature Elimination
RMS	Root Mean Square
RNN	Recurrent Neural Network
ROC	Receiver Operating Characteristic
RVMD	Robust Variational Mode Decomposition
SAMME.R	Stagewise Additive Modeling using a Multiclass Exponential Loss Function
SLBP	Symmetrically Weighted Local Binary Patterns
SpKit	Signal Processing Toolkit
SVM	Support Vector Machine
SZ (SCZ)	Schizophrenia
θ	Theta Frequency Band
α	Alpha Frequency Band
β	Beta Frequency Band
δ	Delta Frequency Band
