# Supplementary material for: Configurable Modular EEG Classification Framework with Multiscale Features and Ensemble Learning: A Reproducible Evaluation for Schizophrenia Detection
Source: Bioengineering (Basel). 2026 Apr 7;13(4):430. doi: 10.3390/bioengineering13040430 (PMC13113903; doi:10.3390/bioengineering13040430)
Supplement: Supplementary file 1 [file bioengineering-13-00430-s001.zip › bioengineering-4166030-supplementary.pdf]

Supplementary Materials

for "Configurable Modular EEG Classification Framework With Multiscale Features and Ensemble Learning: A Reproducible Evaluation for Schizophrenia Detection"

This supplementary section provides detailed performance results for the two schizophrenia EEG datasets analyzed in the study, focusing on conventional epoch-level validation without Leave-One-Subject-Out (LOSO) cross-validation. The tables and figures summarize both class-level and overall metrics, as well as classifier-specific performance.

- Table S1 reports class-level precision, recall, F1-score, and support for Dataset 1.
- Table S2 shows the performance of different classification methods on Dataset 1, including the ensemble model, with hyperparameters tuned via 5-fold cross-validation.
- Table S3 reports class-level precision, recall, F1-score, and support for Dataset 2.
- Table S4 presents the performance of individual classifiers and the ensemble model on Dataset 2, also tuned via 5-fold cross-validation.

The supplementary figures provide visual insights into model performance:

- Figure S1 (Dataset 1) includes (a) the ROC curve with an AUC of 0.998, demonstrating excellent discriminative ability, and (b) precision, recall, and F1-score plotted across classification thresholds, highlighting the optimal threshold at 0.434 where the F1-score is maximized.
- Figure S2 (Dataset 2) shows (a) the ROC curve and (b) precision, recall, and F1-score across thresholds for the proposed model.

These results allow readers to compare conventional cross-validation performance with the more conservative LOSO evaluation reported in the main text, providing a clear benchmark of model generalization under standard and subject-level validation schemes.

S1. Dataset 1 Performance Metrics (Conventional Validation, Without LOSO)

Table S1. Performance Metrics of Dataset 1

Class	Precision	Recall	F1-score	Support
Control	0.966	0.9924	0.979	3292
SZ	0.9935	0.9706	0.9819	3914
Macro Avg	0.9797	0.9815	0.9805	7206
Weighted Avg	0.9809	0.9806	0.9806	7206

Note: These results reflect epoch-level evaluation, where data from the same subject may appear in both training and testing sets. Performance is thus higher than LOSO results, which prevent subject-level data leakage. Recall is class-specific: the SZ recall measures how well the model detects schizophrenia (sensitivity), and the Control recall measures how well healthy subjects are correctly identified (specificity).

Table S2. Performance of Different Classification Methods on Dataset 1

Classification Method	Accuracy (%)	Notes
Support Vector Machine (SVM)	94.52	RBF kernel, tuned via 4-fold CV
K-Nearest Neighbors (KNN)	87.37	Tuned neighbors & weighting
Extremely Randomized Trees	98.75	Best single model
Random Forest	98.68	Tuned depth & estimators
Decision Tree	95.21	Single-tree baseline
AdaBoost	95.56	SAMME.R (deprecated warning noted)
Naive Bayes	65.09	Poor fit to feature distribution
ML Ensemble (Voting)	98.06	Best overall (test set)

Note: These conventional validation results illustrate the inflated performance estimates achievable when subject-level separation is not enforced, reinforcing the necessity of LOSO evaluation for realistic assessment.

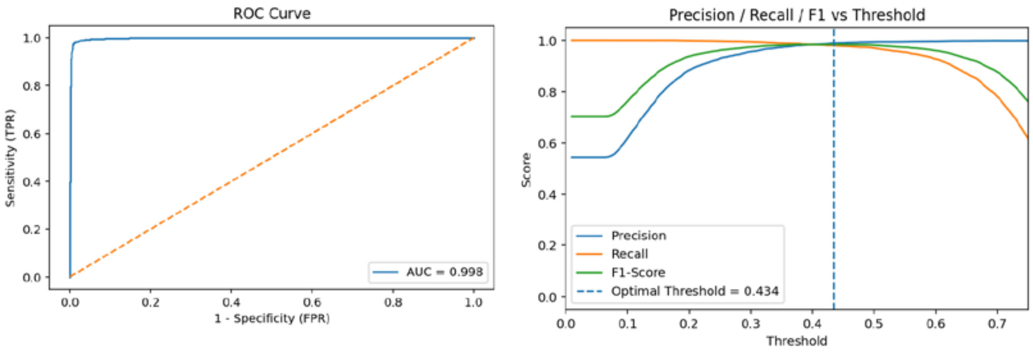

(a) (b)

Figure S1. (a) ROC curve for Dataset 1 showing the trade-off between sensitivity (True Positive Rate) and 1-specificity (False Positive Rate) for the classifier. The curve demonstrates excellent performance with an Area Under the Curve (AUC) of 0.998, indicating high discriminative ability. (b) Precision, Recall, and F1-Score plotted against different classification thresholds for Dataset 1. The plot identifies the optimal threshold at 0.434, where the F1-Score is maximized, balancing precision and recall effectively.

S2. Dataset 2 Performance Metrics (Conventional Validation, Without LOSO)

Table S3. Performance Metrics of Dataset 2

Class	Precision	Recall	F1-score	Support
Control	0.9202	0.8829	0.9011	222
SZ	0.9107	0.9397	0.925	282
Macro Avg	0.9154	0.9113	0.9131	504
Weighted Avg	0.9149	0.9147	0.9145	504

Note: These metrics reflect epoch-level validation, where data from the same subject may appear in both training and testing sets. Performance is therefore higher than what is observed under strict LOSO evaluation, which prevents subject-level data leakage and provides a more realistic estimate of generalization to unseen subjects. Recall is class-specific: the SZ recall measures how well the model detects schizophrenia (sensitivity), and the Control recall measures how well healthy subjects are correctly identified (specificity).

Table S4. Performance of Different Classification Methods on Dataset 2

Classification Method	Accuracy (%)	Notes
Support Vector Machine (SVM)	71.04	Baseline only (below tuning threshold)
K-Nearest Neighbors (KNN)	75.5	Baseline only (below tuning threshold)
Extremely Randomized Trees	88.12	Tuned via 4-fold CV
Random Forest	85.89	Tuned via 4-fold CV
Decision Tree	77.23	Baseline only
AdaBoost	79.46	Baseline only
Naive Bayes	71.29	Baseline only
ML Ensemble (Voting)	91.47	Best overall (test set)

Note: These conventional validation results illustrate the inflated performance estimates achievable without subject-level separation. They serve as a useful reference for comparison to LOSO results, highlighting the critical impact of leakage-free, subject-level evaluation for realistic EEG-based schizophrenia classification.

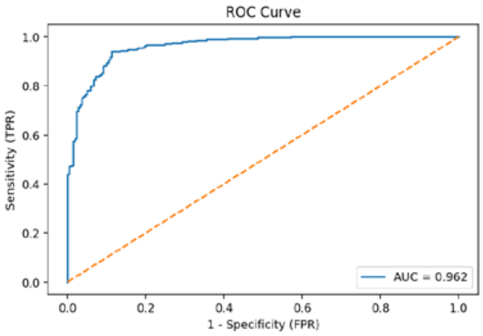

(a)

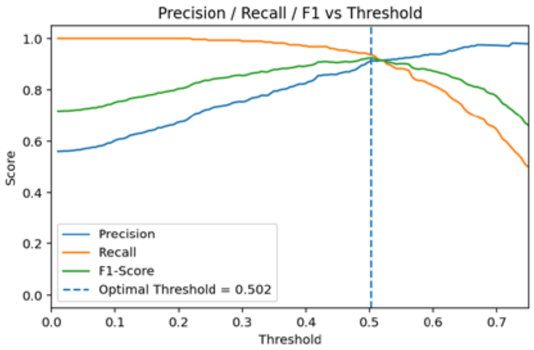

(b)

Figure S2. (a) Receiver Operating Characteristic (ROC) curve for the proposed classification model evaluated on Dataset 2. (b) Precision, recall, and F1-score plotted against classification thresholds for the proposed model on Dataset 2.
